# Supplementary material for: Membrane Lipids Augment Cell Envelope Stress Signaling and Resistance to Antibiotics and Antimicrobial Peptides in Enterococcus faecalis
Source: bioRxiv. 2023 Oct 19:2023.10.17.562839. Preprint. [Version 2] doi: 10.1101/2023.10.17.562839 (PMC10614854; doi:10.1101/2023.10.17.562839)
Supplement: Supplement 1 [file media-1.pdf]

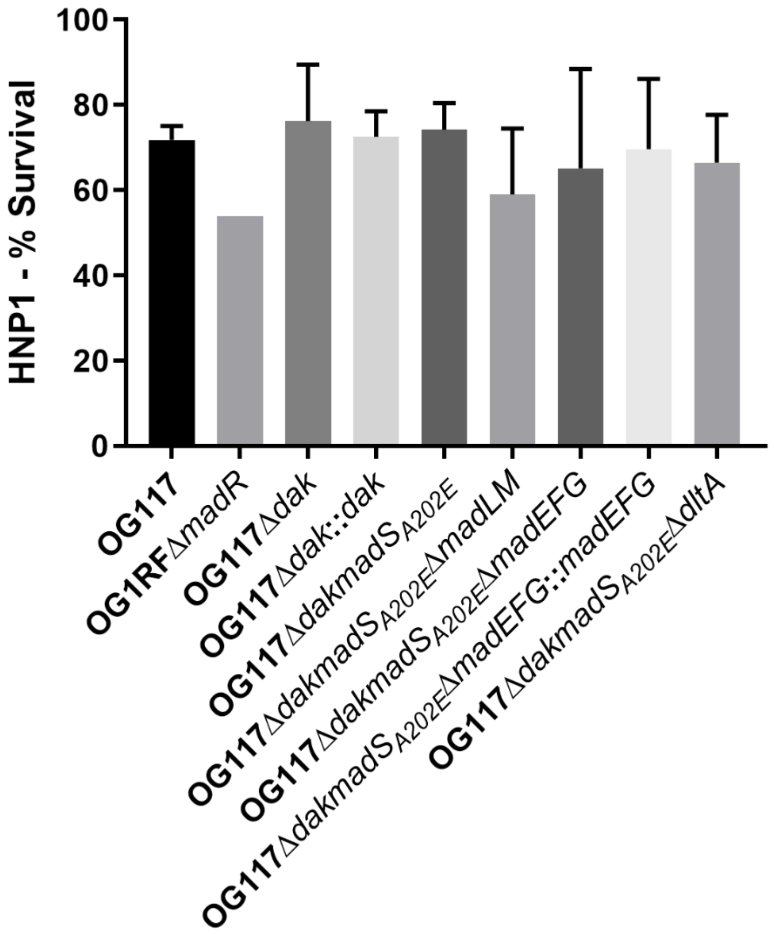

Supplemental Figure 1. HNP1 peptide killing assay. Bacterial isolates were incubated with HNP1 at 10 µg/mL. Percent survival was calculated by dividing the number of colony forming units per milliliter (CFU/mL) after HNP1 exposure by the CFU/mL of assay buffer growth control. No significant differences were seen across any of the strains at the tested conditions, error bars represent standard deviation of three independent runs.

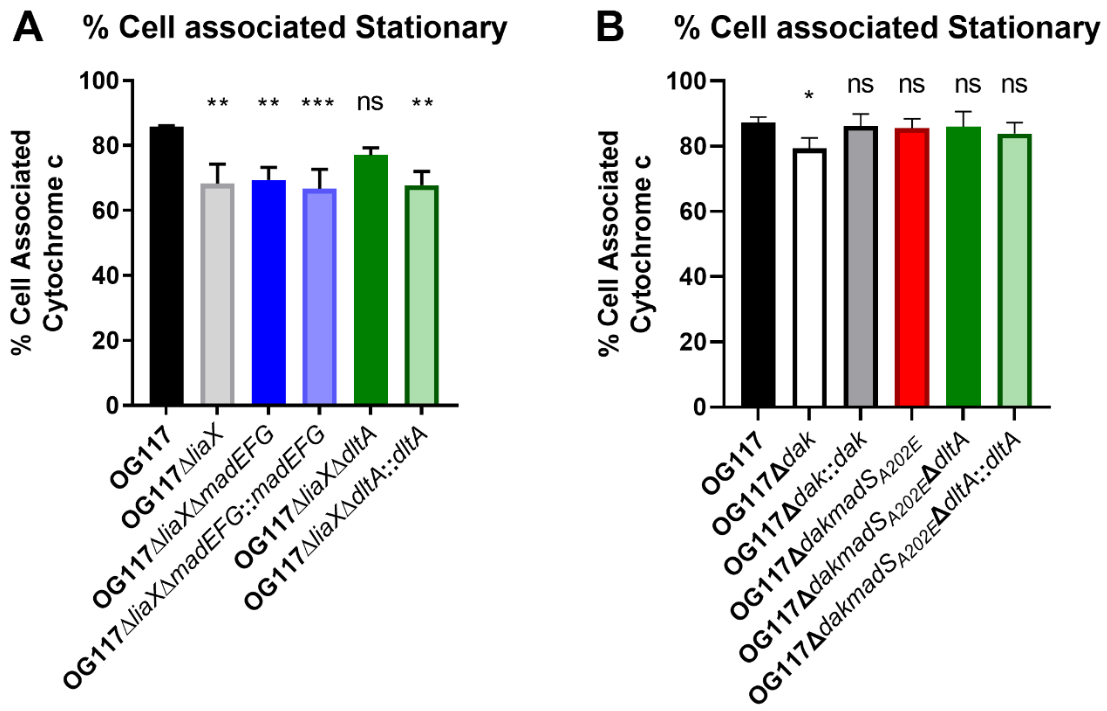

Supplemental Figure 2. Cytochrome c binding assay. Cell surface charge was assessed via binding of the cationic protein cytochrome c to cells in stationary phase. Increased positive cell surface charge (i.e., via Dlt mediated D-alanylation of lipo- and wall-teichoic acids) would decrease cell associated cytochrome c. **(A)** A decrease in cell associated cytochrome c was seen in the OG117Δ*liaX* background as compared to OG117. This difference in surface was abolished on deletion of *dltA* and restored with complementation of *dltA* in the native chromosomal location. **(B)** Deletion of *dak* was associated a significant decrease in cell associated cytochrome c as compared to OG117, however no significant changes were seen in the OG117Δ*dakmadS*$_{A202E}$ background. *, p<0.05; **, p<0.01; ***, p<0.001; ns, not significant. Error bars represent standard deviation of three independent runs.

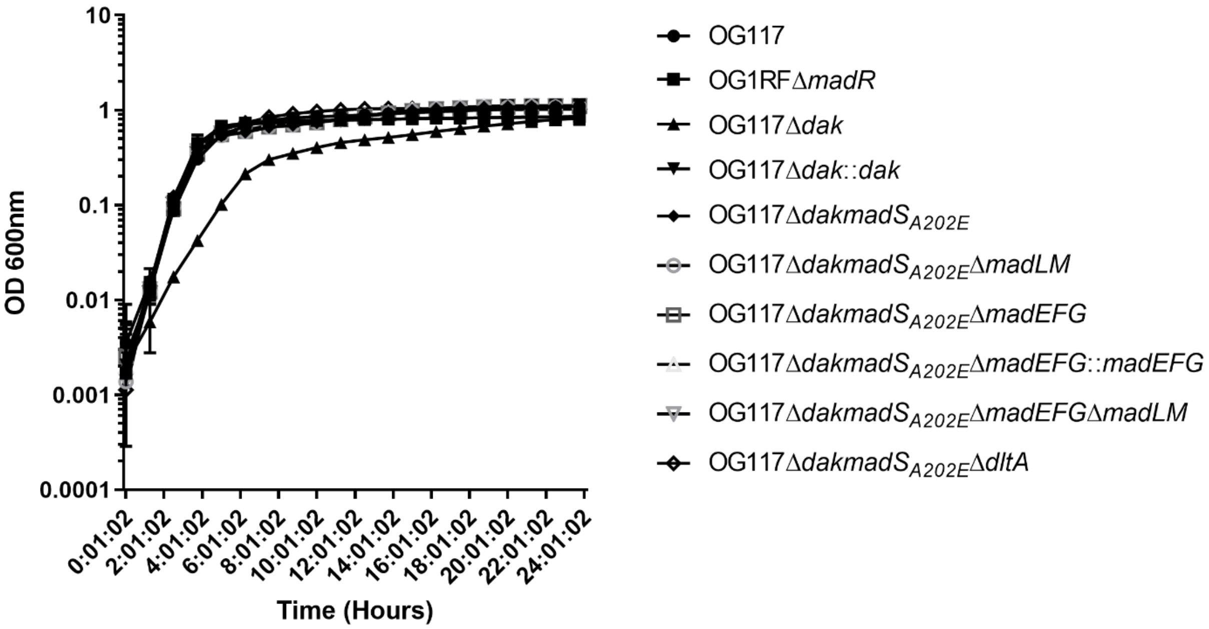

Supplemental Figure 3. Growth curves for selected strains. Bacteria were inoculated at approximately $1x10^6$ CFU/mL and grown in brain heart infusion broth for 24 hours at 37° C in 96 well plate format. Optical density measurements at 600 nm were taken every 15 minutes, every 4th measurement was graphed for clarity. Error bars represent standard deviation of at least 11 replicate wells.

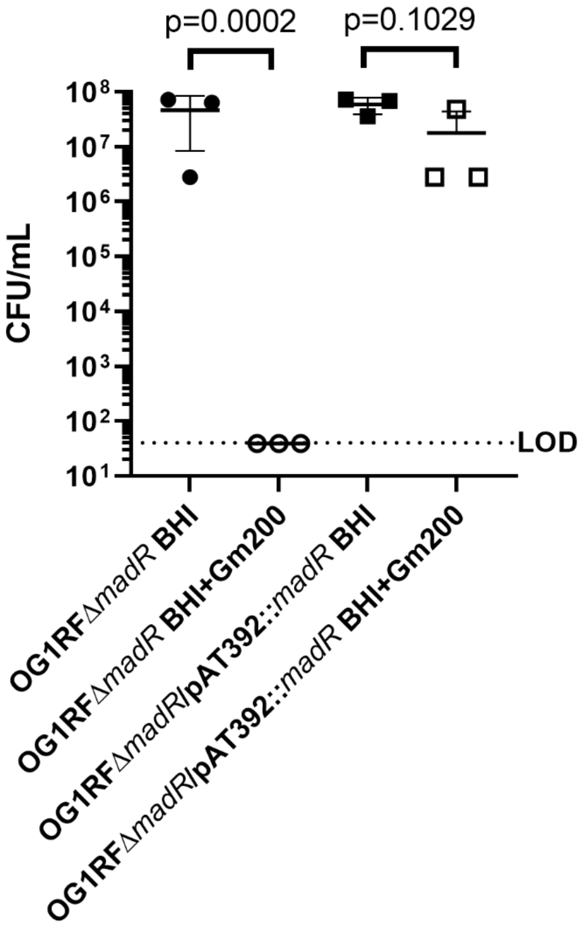

Supplemental Figure 4. Plasmid stability for pAT392 in the absence of gentamicin exposure. The stability of OG1RFΔ*madR* containing the plasmid pAT392::*madR* was determined after overnight passage in antibiotic free BHI media. Ten-fold serial dilutions for each strain were plated on BHI agar and BHI agar containing 200 µg/mL of gentamicin (Gm) for CFU determination. Statistical differences were determined by unpaired t-test of the log-transformed CFU. The limit of detection for the assay was 40 CFU/mL. The assay was performed in triplicate.

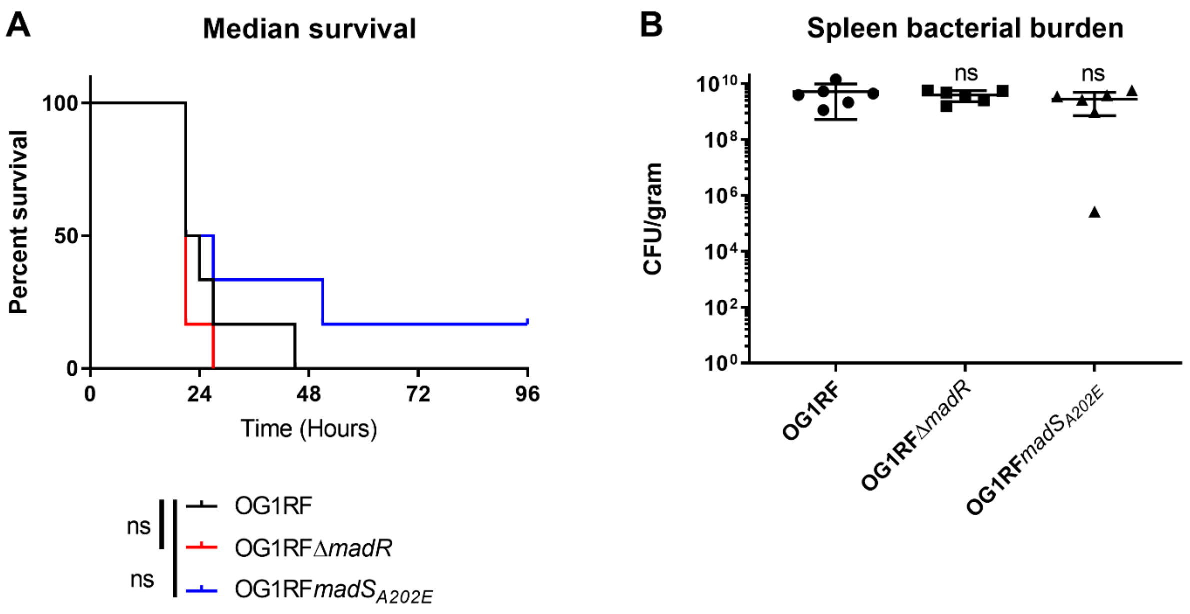

Supplemental Figure 5. *E. faeacalis* mouse peritonitis model. Female outbred ICR mice (n=6 per strain) were inoculated via intraperitoneal injection with approximately $5x10^8$ CFU/mL of bacteria in sterile rat fecal extract. **(A)** Kaplan-Meier survival curves for mice. Statistical differences in median survival were assessed by the log-rank test, there were no differences in median survival between the strains. **(B)** Bacterial burden from the spleen for each strain. Statistical differences were assessed by one-way ANOVA with Tukey's test for multiple comparisons. There were no significant differences between the strains.

**Exponential Phase**	OG117	OG117Δ*dak*	OG117Δ*dak*::*dak*
Phosphatidylglycerol			
PG 30:0	6.07 ± 0.36	4.71 ± 0.09	5.67 ± 0.16
PG 32:0	11.36 ± 0.88	**16.42 ± 1.34**	11.73 ± 0.94
PG 34:1	59.10 ± 1.55	**57.36 ± 1.76**	**56.55 ± 1.73**
PG 35:1	17.00 ± 0.67	**19.23 ± 0.69**	**19.66 ± 0.99**
PG 36:1	4.91 ± 0.21	**1.53 ± 0.07**	4.42 ± 0.2
PG 37:2	1.39 ± 0.06	0.73 ± 0.03	1.79 ± 0.1
PG 38:2	0.19 ± 0.02	0.018 ± 0.005	0.18 ± 0.013
Lysylphosphatidylglycerol			
LysylPG 30:0	3.91 ± 0.7	**1.71 ± 0.29**	4.58 ± 0.65
LysylPG 30:1	2.12 ± 0.13	**0.08 ± 0.11**	2.22 ± 0.2
LysylPG 32:0	6.97 ± 1.48	**9.98 ± 0.35**	8.28 ± 1.63
LysylPG 32:1	13.58 ± 0.82	12.19 ± 1.12	13.12 ± 1.17
LysylPG 34:1	53.94 ± 1.54	**67.67 ± 2.08**	**50.96 ± 1.64**
LysylPG 34:2	4.96 ± 0.57	**1.25 ± 0.29**	3.82 ± 0.5
LysylPG 35:1	8.79 ± 1.29	7.12 ± 0.71	**11.76 ± 1.65**
LysylPG 36:2	6.11 ± 0.53	**0.005 ± 0.01**	5.27 ± 0.45
Cardiolipin			
CL 64:2	10.02 ± 1.53	9.12 ± 0.70	9.14 ± 0.35
CL 65:2	3.24 ± 0.45	3.92 ± 0.3	3.21 ± 0.06
CL 66:2	20.44 ± 1.19	**23.41 ± 0.93**	19.55 ± 0.27
CL 67:2	7.73 ± 0.24	8.23 ± 0.18	8.35 ± 0.15
CL 68:2	28.58 ± 0.38	**33.01 ± 1.26**	27.85 ± 1.05
CL 69:2	11.68 ± 0.34	11.84 ± 0.36	**13.43 ± 0.45**
CL 70:3	12.73 ± 1.66	**7.8 ± 0.68**	12.40 ± 0.82
CL 71:3	3.92 ± 0.52	**1.49 ± 0.18**	4.33 ± 0.47
CL 72:4	1.67 ± 0.61	0.59 ± 0.1	1.75 ± 0.44
Diacylglycerol			
DG 32:0	32.19 ± 1.88	**44.95 ± 0.76**	34.96 ± 5.08
DG 34:1	49.53 ± 1.36	**43.31 ± 0.96**	47.95 ± 4.87
DG 34:2	18.28 ± 1.26	**11.74 ± 0.5**	17.1 ± 0.95
Diglucodiacylglycerol			
DGDG 30:0	2.89 ± 0.12	**4.67 ± 0.18**	3.24 ± 0.2
DGDG 30:1	2.28 ± 0.06	**1.15 ± 0.08**	1.99 ± 0.1
DGDG 32:0	11.95 ± 0.71	**20.64 ± 0.73**	**13.05 ± 1.31**
DGDG 34:1	67.32 ± 0.55	**60.05 ± 0.77**	66.84 ± 0.1
DGDG 35:1	6.93 ± 0.18	**10.35 ± 0.34**	6.9 ± 0.31
DGDG 36:2	8.63 ± 0.36	**3.14 ± 0.095**	8.0 ± 0.51

**Stationary Phase**	OG117	OG117Δ*dak*	OG117Δ*dak*::*dak*
Phosphatidylglycerol			
PG 30:0	11.72 ± 0.28	**7.24 ± 0.24**	11.09 ± 0.16
PG 32:0	11.09 ± 0.46	10.42 ± 0.55	11.11 ± 0.35
PG 34:1	30.91 ± 0.9	**37.78 ± 0.87**	31.59 ± 0.56
PG 35:1	38.47 ± 0.48	**39.68 ± 1.14**	38.45 ± 0.56
PG 36:1	2.99 ± 0.07	**1.94 ± 0.05**	3.07 ± 0.06
PG 37:2	4.72 ± 0.13	**2.9 ± 0.15**	4.6 ± 0.12
PG 38:2	0.09 ± 0.005	0.05 ± 0.001	0.09 ± 0.008
Lysylphosphatidylglycerol			
LysylPG 30:0	13.13 ± 0.38	**5.54 ± 0.18**	12.43 ± 0.37
LysylPG 30:1	5.95 ± 0.24	**3.91 ± 0.29**	5.33 ± 0.15
LysylPG 32:0	8.7 ± 0.49	**5.27 ± 0.37**	9.09 ± 0.38
LysylPG 32:1	14.25 ± 0.5	**16.5 ± 0.58**	13.77 ± 0.31
LysylPG 34:1	24.79 ± 0.71	**33.82 ± 0.69**	**26 ± 0.25**
LysylPG 34:2	1.55 ± 0.19	**3.24 ± 0.36**	1.66 ± 0.26
LysylPG 35:1	29.91 ± 1.56	29.31 ± 0.6	29.84 ± 0.37
LysylPG 36:2	1.75 ± 0.18	2.42 ± 0.3	1.88 ± 0.31
Cardiolipin			
CL 64:2	13.96 ± 0.44	**10.51 ± 0.36**	**13.05 ± 0.91**
CL 65:2	11.32 ± 0.27	**8.91 ± 0.29**	**10.08 ± 0.86**
CL 66:2	17.25 ± 0.4	16.66 ± 0.36	17.27 ± 0.23
CL 67:2	13.37 ± 0.3	**14.42 ± 0.27**	12.89 ± 0.45
CL 68:2	17.82 ± 0.63	**19.27 ± 0.34**	**18.94 ± 1.15**
CL 69:2	12.58 ± 0.29	**14.14 ± 0.28**	13.05 ± 0.6
CL 70:3	9.96 ± 0.25	**12.25 ± 0.3**	10.56 ± 0.28
CL 71:3	2.9 ± 0.2	2.95 ± 0.15	3.27 ± 0.17
CL 72:4	0.84 ± 0.06	0.89 ± 0.18	0.9 ± 0.1
Diacylglycerol			
DG 32:0	41.14 ± 0.3	**43.88 ± 1.38**	**45.12 ± 2.08**
DG 34:1	43.64 ± 0.6	43.35 ± 1.38	**39.16 ± 1.19**
DG 34:2	15.22 ± 0.82	**12.78 ± 0.25**	15.72 ± 0.9
Diglucodiacylglycerol			
DGDG 30:0	5.25 ± 0.23	5.91 ± 0.1	5.44 ± 0.24
DGDG 30:1	2.47 ± 0.03	1.61 ± 0.2	2.38 ± 0.04
DGDG 32:0	12.04 ± 0.31	**13.17 ± 0.18**	12.44 ± 0.62
DGDG 34:1	59.03 ± 0.9	**51.66 ± 0.57**	58.95 ± 1.23
DGDG 35:1	13.85 ± 0.88	**23.08 ± 0.64**	13.7 ± 0.78
DGDG 36:2	7.36 ± 0.24	**4.57 ± 0.14**	7.1 ± 0.38

Supplemental Table 2. Membrane lipid composition of E. faecalis OG117 strains. Percent fatty acyl chain composition for each lipid species. Bold values indicate significant difference as compared to OG117 ($p \leq 0.05$, two-way ANOVA, with Tukey's test for multiple comparisons).

Supplemental Table 3. Strains and plasmids used in this study.

Strain/Plasmid	Relevant Characteristics	Ref
E. faecalis		
OG1RF	Laboratory strain of *E. faecalis*, WGS accession CP002621.1	[1]
OG1RFΔ*madR*	Non-polar deletion of *madR* in OG1RF wild type background	[2]
OG1RF*madSA202E*	OG1RF with exchange of the *madS* gene for the allele with a C>A nucleotide change at position 605, resulting in the amino acid change A202E	This study
OG1RFΔ*liaR*	Non-polar deletion of *liaR* in OG1RF wild type background	[3]
OG1RFΔ*liaRmadSA202E*	OG1RFΔ*liaR* with exchange of the *madS* gene for the allele with a C>A nucleotide change at position 605, resulting in the amino acid change A202E	This study
OG117	Derivative of OG1RF with the *E. faecalis* ATCC 4200 CRISPR1 *cas9* gene inserted in a neutral genomic insertion site	[4]
OG117Δ*dak*	Derivative of OG117 with a deletion of dak gene (OG1RF_12374) leaving only first 13 amino acids and 6 amino acids just prior to stop codon (Met N V T E I S A G Q F Q E V F V M K K Stop)	This study
OG117Δ*dak::dak*	Complementation of OG117Δ*dak* with the full length dak gene in the native chromosomal location	This study
OG117Δ*dakmadSA202E*	Derivative of OG117Δ*dak* with *madSA202E* allele, contains ser->tyr change in FabT at amino acid 36 in the DNA binding domain	This study
OG117Δ*dakmadSA202E*Δ*madLM*	Derivative of OG117Δ*dakmadSA202E* with deletion of *madLM* (OG1RF_11656 and OG1RF_11657) encoding the MadLM ABC transporter, contains ser->tyr change in FabT at amino acid 36 in the DNA binding domain	This study
OG117Δ*dakmadSA202E*Δ*madEFG*	Derivative of OG117Δ*dakmadSA202E* with deletion of *madEFG* (OG1RF_12267, OG1RF_12268, and OG1RF_12269), contains ser->tyr change in FabT at amino acid 36 in the DNA binding domain	This study
OG117Δ*dakmadSA202E*Δ*madEFG*::*madEFG*	Derivative of OG117Δ*dakmadSA202E*Δ*madEFG* with complementation of *madEFG* in the native chromosomal location, contains ser->tyr change in FabT at amino acid 36 in the DNA binding domain	This study
OG117Δ*dakmadSA202E*Δ*madEFG*Δ*madLM*	Derivative of OG117Δ*dakmadSA202EmadEFG* with deletion of *madLM*, contains ser->tyr change in FabT at amino acid 36 in the DNA binding domain	This study
OG117Δ*dakmadSA202E*Δ*dltA*	Derivative of OG117Δ*dakmadSA202E* with deletion of *dltA* (OG1RF_12112), contains	This study

	ser->tyr change in FabT at amino acid 36 in the DNA binding domain	
OG117Δ*dakmadSA202E*Δ*dltA*::*dltA*	Derivative of OG117Δ*dakmadSA202EdltA* with complementation of *dltA* in the native chromosomal location, contains ser->tyr change in FabT at amino acid 36 in the DNA binding domain	This study
OG117Δ*liaX*	Derivative of OG117 with deletion of the *liaX* gene	[5]
OG117Δ*liaX*Δ*madEFG*	Derivative of OG117Δ*liaX* with deletion of *madEFG*	This study
OG117Δ*liaX*Δ*madEFG::madEFG*	Derivative of OG117Δ*liaX*Δ*madEFG* with complementation of *madEFG* in the native chromosomal location	This study
OG117Δ*liaX*Δ*dltA*	Derivative of OG117Δ*liaX* with deletion of *dltA*	This study
OG117Δ*liaX*Δ*dltA::dltA*	Derivative of OG117Δ*liaX*Δ*dltA* with complementation of *dltA* in the native chromosomal location	This study
Plasmids		
pHOU1	Derivative of pCJK47 in which the *erm*(C) gene was replaced by *aph-2”-ID*; confers GEN resistance	[6]
pCE	*oriT* from pCF10, containing the constitutive P_{bacA} promoter from pPD1 to express the guide RNA, *cat* for chloramphenicol selection, and *pheS** for p-chloro-phenylalanine counterselection	[4]
pAT392	$oriR_{\text{pAM}\beta1}$, $oriR_{\text{pUC}}$ $oriT_{\text{RK2}}$ *spc lacZ*α P2 *aac(6’)-aph(2”)*	[7]
pAT392::*madR*	Derivative of pAT392 with *madR* and upstream sequence containing ribosomal binding site	[2]

Supplemental Table 4. Primers used in this study.

Primer	Sequence	Notes
dltA_spacer_F	GTAATTAATATGATTCAAACGATTGATGAAgtt ttagagtcatgttgtttagaatgg	forward primer for *dltA* targeting guide RNA spacer
dltA_spacer_R	TTCATCAATCGTTTGAATCATATTAATTACtttc attgctattatacccatgtag	reverse primer for *dltA* targeting guide RNA spacer
pCE_dltA_AF	atattacagctccagatccatatccttcttCCGAAACTTG GCGGCTAAAT	Forward primer for cloning upstream crossover region of *dltA* into pCE
dltA_linker_AR	GCCGCAATTAGCAGAACGTATAGCCGCCTCCT TAAAACTC	Reverse primer for cloning upstream crossover region of *dltA* into pCE
dltA_linker_BF	GAGGCGGCTATACGTTCTGCTAATTGCGGCCT TG	Forward primer for cloning downstream crossover region of *dltA* into pCE
pCE_dltA_BR	gaagcgaaaaaggagaagtcggttcagaaaCCAACTT AGGAACGGTTTGTTAAAG	Reverse primer for cloning downstream crossover region of *dltA* into pCE
dltA_Ext_F	CAAATGCCTAACGAAGAGGC	Screening primer for *dltA* mutants, anneals to gDNA external to crossover region upstream of the gene
dltA_Int_F	CTGTAAAAGCCGTTTTGAAGC	Screening primer for *dltA* mutants, anneals to gDNA internal to crossover region upstream of the gene
dltA_Int_R	CCCTAAAAAGCCAAGTAAGGTTG	Screening primer for *dltA* mutants, anneals to gDNA internal to crossover region downstream of the gene
dltA_Ext_R	GATAAGGTCATGTGCCAACG	Screening primer for *dltA* mutants, anneals to gDNA external to crossover region downstream of the gene
yxdL_spacer_F	TTTATTGGCAACAATCGATAGTCCAACAGAgtt ttagagtcatgttgtttagaatgg	forward primer for *madL* targeting guide RNA spacer
yxdL_spacer_R	TCTGTTGGACTATCGATTGTTGCCAATAAAtttc attgctattatacccatgtag	reverse primer for *madL* targeting guide RNA spacer
pCE_yxdLM_AF	atattacagctccagatccatatccttcttAGAAGAAAG AGCGTGCTTTA	Forward primer for cloning upstream crossover region of *madLM (yxdLM)* into pCE
yxdLM_linker_AR	GCTTAACGATTTTTTATAAATTTTCCACTCCTA TTCTTCTC	Reverse primer for cloning upstream crossover region of *madLM (yxdLM)* into pCE

yxdLM_linker_BF	GGAGTGGAAAATTTATAAAAAATCGTTAAGCATGCAC	Forward primer for cloning downstream crossover region of *madLM (yxdLM)* into pCE
pCE_yxdLM_BR	gaagcgaaaaaggagaagtcggttcagaaaCGAACTTCGTTGTTAGGAGC	Reverse primer for cloning downstream crossover region of *madLM (yxdLM)* into pCE
yxdLM_EF	CTTAGTAGATGAATTGGAAGCAC	Screening primer for *madLM (yxdLM)* mutants, anneals to gDNA external to crossover region upstream of the gene
yxdLM_IF	CACCTTTCACGGTTATATCTAATCG	Screening primer for *madLM (yxdLM)* mutants, anneals to gDNA internal to crossover region upstream of the gene
yxdLM_IR	CAGATAATCCATATTCTTGACGC	Screening primer for *madLM (yxdLM)* mutants, anneals to gDNA internal to crossover region downstream of the gene
yxdLM_ER	CCTGCACAAATGGTTAAGGC	Screening primer for *madLM (yxdLM)* mutants, anneals to gDNA external to crossover region downstream of the gene
EF2987_spacer_F	TCAGGCCAGAAGAAGCGAAATAAAAAAGTGgttttagagtcatgttgtttagaatgg	forward primer for *madG* targeting guide RNA spacer
EF2987_spacer_R	CACTTTTTTATTTCGCTTCTTCTGGCCTGAtttcattgctattatacccatgtag	reverse primer for *madG* targeting guide RNA spacer
pCE_EF2987_AF	atattacagctccagatccatatccttcttTGCAGCGATGAACCTTGTAGA	Forward primer for cloning upstream crossover region of *madEFG* into pCE
EF2987_linker_AR	GTTTCTTTGCTGTTTATATTTATTTGTTCCTCCTTGATTTCTGTTGAC	Reverse primer for cloning upstream crossover region of *madEFG* into pCE
EF2987_linker_BF	GAGGAACAAATAAATATAAACAGCAAAGAAACAGCCATTTTTG	Forward primer for cloning downstream crossover region of *madEFG* into pCE
pCE_EF2987_BR	gaagcgaaaaaggagaagtcggttcagaaaTTCTGCGGCTGTTAGCTTTATTC	Reverse primer for cloning downstream crossover region of *madEFG* into pCE
EF2987_Ext_F	GAGTTTACCTATGCGCCGCC	Screening primer for *madEFG* (EF2987-EF2985) mutants, anneals to gDNA external to crossover region upstream of the gene
EF2987_Int_F	GTGCGTTTCTCAGTCAACAAGG	Screening primer for *madEFG* (EF2987-EF2985) mutants, anneals to gDNA internal to crossover region upstream of the gene

EF2987_Int_R	CAGTCATTCCTGTAATCCCCGA	Screening primer for *madEFG* (EF2987-EF2985) mutants, anneals to gDNA internal to crossover region downstream of the gene
EF2987_Ext_R	GAGGTCATCCTGTGATGGTG	Screening primer for *madLM madEFG* (EF2987-EF2985) mutants, anneals to gDNA external to crossover region downstream of the gene
DAK_comp_spacer_F	CGTTTTTATTTTTTCATCACGAAAACTTCCgttttagagtcatgttgtttagaatgg	forward primer for *dak* targeting guide RNA spacer
DAK_comp_spacer_R	GGAAGTTTTCGTGATGAAAAAATAAAAACGtttcattgctattatacccatgtag	reverse primer for *dak* targeting guide RNA spacer
delDAK2_DownF_NotI	GGGCGGCCGCAAGCGATCCACGTCATATTG	Forward primer for cloning downstream crossover region of *dak* into pCE, NotI restriction site underlined
delDAK2_DownR	GTGTATCCATACTTATTCTCAGCAGAATAG	Reverse primer for cloning downstream crossover region of *dak* into pCE
delDAK2_UpF	TTCCTGGAACTGACCTGC	Forward primer for cloning upstream crossover region of *dak* into pCE
delDAK2_UpR_PstI	GGCTGCAGCATTCGGTCTGTAGATATGGC	Reverse primer for cloning upstream crossover region of *dak* into pCE, PstI restriction site underlined
delDAK2_ExtDownF	GCATATGCTTCGGACTTCGC	Screening primer for *dak* mutants, anneals to gDNA external to crossover region downstream of the gene
delDAK2_IntDownF	TCGAAACGTTAACCGGCTCT	Screening primer for *dak* mutants, anneals to gDNA internal to crossover region downstream of the gene
delDAK2_IntUpR	TCGCAGTGGATGTCTACACG	Screening primer for *dak* mutants, anneals to gDNA internal to crossover region upstream of the gene
delDAK2_ExtUpR	TAAAGTGGAAGCGCTGGACTA	Screening primer for *dak* mutants, anneals to gDNA external to crossover region upstream of the gene
BamHI_2987Comp_up_F	GACGGATCCGATCCTGTGAACATGTTAGGC	Forward primer for cloning *madEFG* complementation into pHOU1, BamHI restriction site underlined

EcoRI_2987Comp_Down_R	GACGAATTCCTATACAAGACAGCCAGTAGTGC	Reverse primer for cloning *madEFG* complementation into pHOU1, EcoRI restriction site underlined
BamHI_2049Comp_Up_F	GACGGATCCAGAAGAAAGAGCGTGCTTTA	Forward primer for cloning *madLM* complementation into pHOU1, BamHI restriction site underlined
BamHI_2049Comp_Down_R	CAGGGATCCCGAACTTCGTTGTTAGGAGC	Reverse primer for cloning *madLM* complementation into pHOU1, BamHI restriction site underlined
dltA_EcoRI_F	GACGAATTCCCGAAACTTGGCGGCTAAAT	Forward primer for cloning *dltA* complementation into pHOU1, EcoRI restriction site underlined
dltA_BamHI_R	GACGGATCCCCAACTTAGGAACGGTTTGTTAAAG	Reverse primer for cloning *dltA* complementation into pHOU1, BamHI restriction site underlined
2050_F	GGTGACCCATGATCCGCTAG	qRT-PCR primer, *madL*
2050_R	TCGACACCTTCAATCTCAGCC	qRT-PCR primer, *madL*
2752_F	ACTGGCTCGCTGGATTCAAA	qRT-PCR primer, *madA*
2752_R	TGTACGGGTCATGTGTCACC	qRT-PCR primer, *madA*
2749_F	GCGCATCGAAAGCTGGTTTT	qRT-PCR primer, *dltA*
2749_R	ACTTCGGTGGCTAACTCAGG	qRT-PCR primer, *dltA*
2987_F	CATCGCTGTACCGCAAAAGC	qRT-PCR primer, *madG*
2987_R	TTTTCGCTTTGCCCGCTTTG	qRT-PCR primer, *madG*
gyrB_F	AAAAGGCATGTTGGCTTCAAA	qRT-PCR primer, DNA gyrase housekeeping gene
gyrB_R	GCTTCCCTGGCAAGTTGCTA	qRT-PCR primer, DNA gyrase housekeeping gene

References:

1 Bourgogne, A. *et al.* Large scale variation in *Enterococcus faecalis* illustrated by the genome analysis of strain OG1RF. *Genome Biol* **9**, R110, doi:10.1186/gb-2008-9-7-r110 (2008).

2 Miller, W. R. *et al.* LiaR-independent pathways to daptomycin resistance in *Enterococcus faecalis* reveal a multilayer defense against cell envelope antibiotics. *Mol Microbiol* **111**, 811-824, doi:10.1111/mmi.14193 (2019).

3 Reyes, J. *et al.* A liaR deletion restores susceptibility to daptomycin and antimicrobial peptides in multidrug-resistant *Enterococcus faecalis*. *J Infect Dis* **211**, 1317-1325, doi:10.1093/infdis/jiu602 (2015).

4 Hullahalli, K., Rodrigues, M., Nguyen, U. T. & Palmer, K. An Attenuated CRISPR-Cas System in *Enterococcus faecalis* Permits DNA Acquisition. *mBio* **9**, doi:10.1128/mBio.00414-18 (2018).

5 Khan, A. *et al.* Antimicrobial sensing coupled with cell membrane remodeling mediates antibiotic resistance and virulence in *Enterococcus faecalis*. *Proc Natl Acad Sci U S A*, doi:10.1073/pnas.1916037116 (2019).

6 Panesso, D. *et al.* The hylEfm gene in pHylEfm of *Enterococcus faecium* is not required in pathogenesis of murine peritonitis. *BMC Microbiol* **11**, 20, doi:10.1186/1471-2180-11-20 (2011).

7 Arthur, M., Depardieu, F., Snaith, H. A., Reynolds, P. E. & Courvalin, P. Contribution of VanY D,D-carboxypeptidase to glycopeptide resistance in *Enterococcus faecalis* by hydrolysis of peptidoglycan precursors. *Antimicrob Agents Chemother* **38**, 1899-1903, doi:10.1128/AAC.38.9.1899 (1994).
